# Bromodomain and BET family proteins as epigenetic targets in cancer therapy: their degradation, present drugs, and possible PROTACs

**DOI:** 10.1039/d0ra07971e

**Published:** 2020-12-24

**Authors:** Mohd. Muddassir, Kunjal Soni, Chetan B. Sangani, Abdullah Alarifi, Mohd. Afzal, Naaser A. Y. Abduh, Yongtao Duan, Poonam Bhadja

**Affiliations:** Department of Chemistry, College of Science, King Saud University Riyadh 11451 KSA; Shri Maneklal M. Patel Institute of Sciences and Research, Kadi Sarva Vishwavidyalaya University Gandhinagar Gujarat 382024 India; Henan Provincial Key Laboratory of Children's Genetics and Metabolic Diseases, Zhengzhou Children's Hospital, Zhengzhou University Zhengzhou 450018 China; Arthropod Ecology and Biological Control Research Group, Ton Duc Thang University Ho Chi Minh City Vietnam; Faculty of Environment and Labour Safety, Ton Duc Thang University Ho Chi Minh City Vietnam poonam.bhadja@tdtu.edu.vn

## Abstract

Alteration in the pattern of epigenetic marking leads to cancer, neurological disorders, inflammatory problems *etc.* These changes are due to aberration in histone modification enzymes that function as readers, writers and erasers. Bromodomains (BDs) and BET proteins that recognize acetylation of chromatin regulate gene expression. To block the function of any of these BrDs and/or BET protein can be a controlling agent in disorders such as cancer. BrDs and BET proteins are now emerging as targets for new therapeutic development. Traditional drugs like enzyme inhibitors and protein–protein inhibitors have many limitations. Recently Proteolysis-Targeting Chimeras (PROTACs) have become an advanced tool in therapeutic intervention as they remove disease causing proteins. This review provides an overview of the development and mechanisms of PROTACs for BRD and BET protein regulation in cancer and advanced possibilities of genetic technologies in therapeutics.

## Abstract 2

Alteration in the pattern of epigenetic marking leads to cancer, neurological disorders, inflammatory problems *etc.*

## Graphical abstract

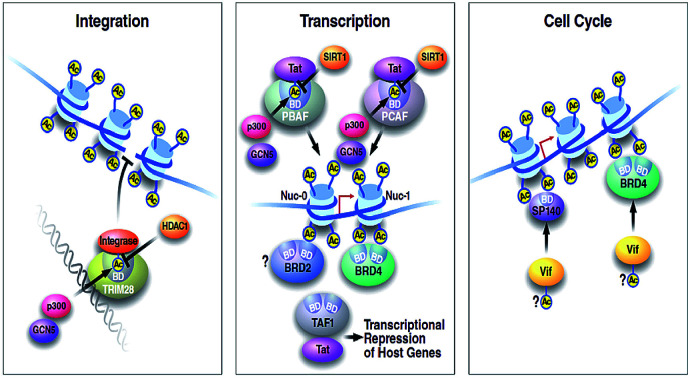

## Introduction

Malignant tumours have been a leading global threat to human health for several decades. Research suggests that approximately 20 million new cases of cancer will be diagnosed every year.^1^ Notable improvements have been recorded in the field of cancer therapy which include inhibition and inhibitors, monoclonal antibodies, and immunotherapies. Small molecule inhibitors could bind tightly to the target protein to inhibit the enzyme activities and induce cell cycle arrest or apoptosis. However, a target protein within tumour cells tends to restore its activity which leads to acquiring drug resistance by overexpressing or mutations in the target protein.^2^ Antibody therapies are more and more popular with the advantage of prolonged pharmacokinetic profile and high binding affinity to targets. The main therapeutic route for antibodies is to interrupt the interaction between extracellular protein and protein or ligand. Also, a series of challenges that have to face include poor membrane permeability, enteral administration, and high cost.^3–5^ RNA interfering molecules often achieve exciting activity to their target protein. Given the catalytic nature, RNAi could work at low exposures because of each siRNA molecule degrading a lot of mRNA transcripts. The shortcomings of current RNAi therapy not only include a lack of oral bioavailability but also poor PK.^6^ And more, it must be pointed out that the treatment of cancer needs a range of therapeutic strategies. A desirable molecule would be with several combined advantages from the small molecule, RNAi modalities, and antibody such as high selectivity, oral bioavailability, and distributing well into the central nervous system (CNS).^7^ In the past two decades, more and more researchers have devoted themselves to exploring an effective therapeutic strategy by the regulation of protein levels to modulate protein function. Some small molecules that control protein expression levels instead of affecting protein function have recently been brought into focus. There is no doubt that the most representative compounds of this kind of molecules are proteolysis-targeting chimeric molecules (PROTAC).^2,8–11^

PROTAC is a strategy to target specific proteins and induce their intracellular degradation. Professor Cruise of Yale University was one of the pioneers in the field related to PROTAC.^12^ Protein knockout induced by PROTAC technology displayed unique advantages over traditional drugs.^13^ On the one hand, PROTACs may achieve higher potency and efficacy compared to traditional small molecule drugs *in vivo*.^14^ Small molecule inhibitors bind to target proteins to achieve an ideal level of therapeutic effects that often-required higher doses and sustained exposure to the target. In contrast, a low dosage of PROTAC could induce tumour regression because of its mechanism which is chemical knockdown rather than by inhibition. On the flip side, the bright calibre to conquer drug resistance that originated through mutation in amino acid binding site.^15^

**Fig. 1:**
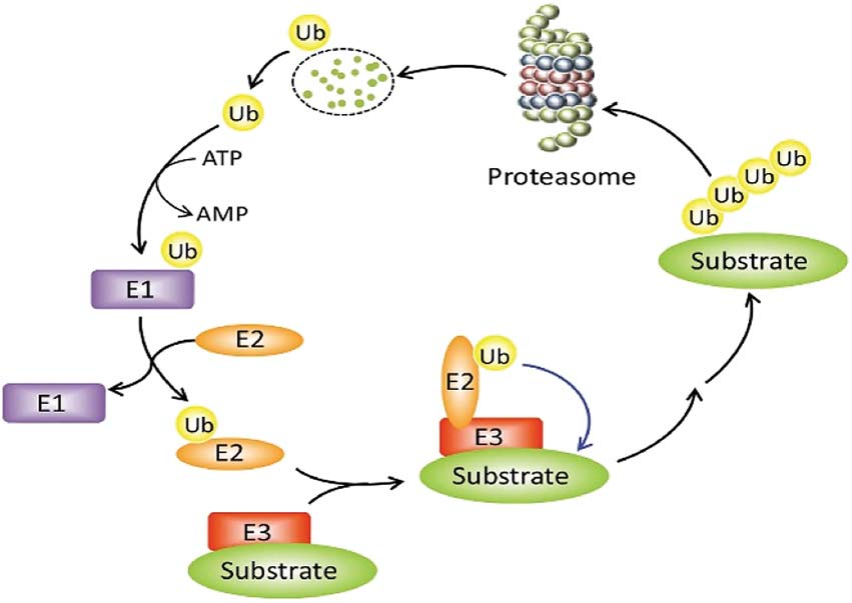
The ubiquitin–proteasome system.

The constancy of the intracellular domain is maintained in many ways. One of the main ways is the ubiquitin protease system (UPS, Fig. 1) where PROTAC could bring about targeted protein degradation.^10^

Ubiquitin, ubiquitin-activating enzyme (E1), ubiquitin-conjugating enzyme (E2), and a ubiquitin ligase (E3), proteasome, target proteins all together develop UPS.^16^ The UPS has an essential role in several vital processes as the protein targets may be cell cycle and apoptosis regulators, transcription factors that regulate cell division and differentiation, growth, signal transduction, and stress response.^17^

Within the UPS a polypeptide that acts as a molecular label, ubiquitin (Ub) has 76 amino acids in length.^18^ With seven lysine residues, each UB polypeptide is interacting in multiple Ub polypeptides linking that ultimately forms a chain of polyubiquitin.^19^ The final fate of protein is decided by the dissemination of ubiquitination patterns, for example, endocytosis, protein sorting, nuclear export of proteins, DNA repair, and transcription regulation have been connected with mono-ubiquitination. Polyubiquitination has been linked to protein degradation, DNA repair, kinase activation, and transcription factor activation.

The formation of protein ubiquitination incorporates three basic modes. Firstly, an E1 ubiquitin-activating enzyme activates Ub at its C-terminus. In the second step, an E2 ubiquitin-conjugating enzyme does conjugation of Ub and in the final third step, an E3 ubiquitin ligase transfers Ub to the substrate protein.^20–22^

For initiation of proteasomal degradation of a target protein, Ub is one of the vital appliances, although ubiquitin-independent mechanisms have also been reported.^23^

In 2000, Zhou *et al.* narrated that by engineered E3 ligases, stable cellular proteins can be degraded in yeast as well as in mammalian cells that leads to PROTAC development.^24^

Recently, PROTAC has been utilized and developed to target epigenetic proteins. DNA methylation, histone modifications, and chromatin remodelling like epigenetic processes have been affected through many environmental and genetic factors that furnish disease progression.^25,26^ These processes are being targeted for unique drug development through epigenetic enzymes, known as readers, writers, and erasers.^27^ Epigenetic investigation and experimentation have tremendous potential in the development of remedy in broad-spectrum disease and oncology. Many small molecules controlling epigenetic mechanisms are known as promising therapeutic agents. Different epigenetic mechanisms have been explored in the past decades such as covalent modifications, RNA transcripts, and nucleosome positioning.^28–30^ Enzymatic chemical modification or recognition of DNA/histone proteins including regarded as the representative of covalent modifications play central roles in many types of epigenetic inheritance. Several epigenetic protein inhibitors such as vorinostat and azacytidine have been approved for cancer treatment by the FDA.^31,32^ Except for epigenetic inhibitors, some PROTAC target epigenetic proteins have been reported by hijacking the UPS which may be an efficient strategy.^33,34^

**Fig. 2:**
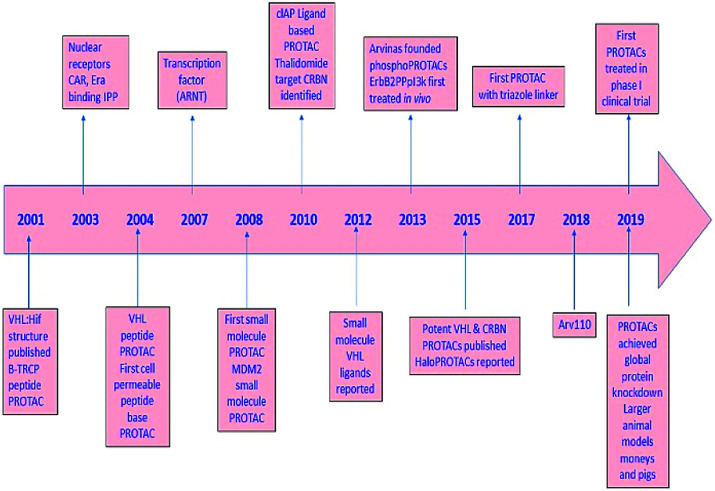
A diagram to demonstrate the development of PROTAC.

PROTAC technology; its development and progress in contrast to epigenetic targets, further scope, and provocation of this advanced passage in the application for treatment are highlighted in the current review (Fig. 2).

### BET protein family

In an organism, many different cells present with the same DNA sequences but they are programmed in such a way that they can do distinctive biological functions and retain different phenotypes for the same. The process is identified as cell differentiation and can be obtained through epigenetics.^35,36^

The structurally flexible N- and C-termini of the core histone octamers within chromatin extended out form nucleosomes. They have vast possibilities of post-translational modifications.^37^ In addition to alterations in DNA methylation, histones with covalent modification are vital apparatus for the epigenetic panorama. Phosphorylation, acetylation, methylation, ubiquitination, and SUMOylation are several kinds of modifications that can be available on histones.^37–39^

In the cell, for genomic stability integration and gene expression or repression, these sites, and state-specific alterations may act conjointly.^39–41^

In the human genome changes in DNA and histone proteins comprising chromatin structure are intently connected with gene transcriptional activation or repression. The post-translational modifications of DNA-packaging histones available with the chromatin shaped this complicated and firmly harmonized alliance.^42^

In cancer, the normal pattern of histone modifications is altered under enzyme deregulations that modify addition, removal, or alters identification of histone markings and mutations.^43^

The information about ∑-N-acetylation of lysine residues (Kac) on histone tails is connected with an open chromatin engineering and transcriptional activation,^44^ despite several acetylation marks that have been correlated in place of chromatin compaction^45^ and with other mechanisms such as, DNA repairing, protein–protein interactions, protein stability, and metabolism^46^ was discovered about 30 years back.

The highly vigorous alteration, lysine acetylation mainly affects chromatin structure and function and even gene transcription.^47–49^ Besides, acetylation of lysine has not been limited to histones but it can also be there on various kinds of transcription-associated proteins, which include histone altering enzymes, transcription factors along with chromatin regulators indicating that it may impact as more common protein function regulators above transcriptional governance agnate to phosphorylation.^50–52^ Acetylation of lysine is one of the essential alterations taking place in histone tails and ambiance of histone code has been extensively investigated.^53^

Histone acetyltransferases (HATs) and histone deacetylases (HDACs) regulate ∑-N-acetylation of lysine molecules at the amino-terminal end of histones. The previous one is known as “writer”, as it does the addition of acetyl group, while the latter has the function of removing acetyl markings, known as ‘erasers'. In cancers, these enzymes are definitely present having the discomfort of mutations and have chances of other free trade mechanisms.^53^

**Fig. 3:**
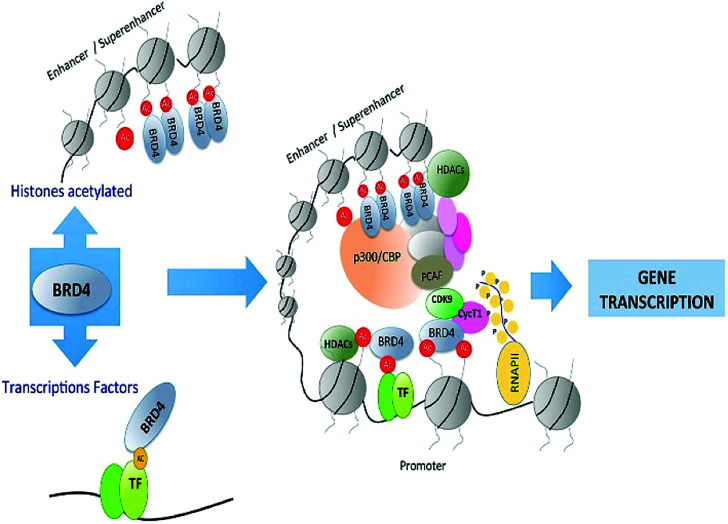
Overview of bromodomain inhibition.

The regulation of gene transcription has been done by Bromodomains (BRDs) as they are recognizing this acetyl marking present in histone tails that have been targeted by chromatin-modifying enzymes and other proteins that are site-specific for chromatin.^53^ Bromodomains (BRDs) are known as “readers” as recognizing this acetyl marking in histone tails (Fig. 3).^53^

‘Readers’ of epigenetic marks are structurally diverse proteins each possessing one or more evolutionarily conserved effecter modules, which recognize covalent modifications of histone proteins or DNA.^54^

The ∑-N-acetylation of lysine molecules can only be specifically verified by Bromodomains (BRD).^54^

The Bromo- and Extra-terminal (BET) family of proteins, including the ubiquitously expressed BRD2, BRD3, and BRD4 and the testis-specific BRDT, recruit transcriptional regulatory complexes to acetylated chromatin thereby controlling specific networks of genes involved in cellular proliferation and cell cycle progression.^55^

Alterations in regulation of activities from BET protein, especially BRD4, have been greatly allied with cancer and inflammatory diseases. This makes BET protein as an appealing drug targets.^56^

### Bromodomains

The histone tails having ∑-N-acetylation on lysine molecules have been determined by bromodomains (BDs), as they perform work of reading of acetylated lysine molecules.

**Fig. 4:**
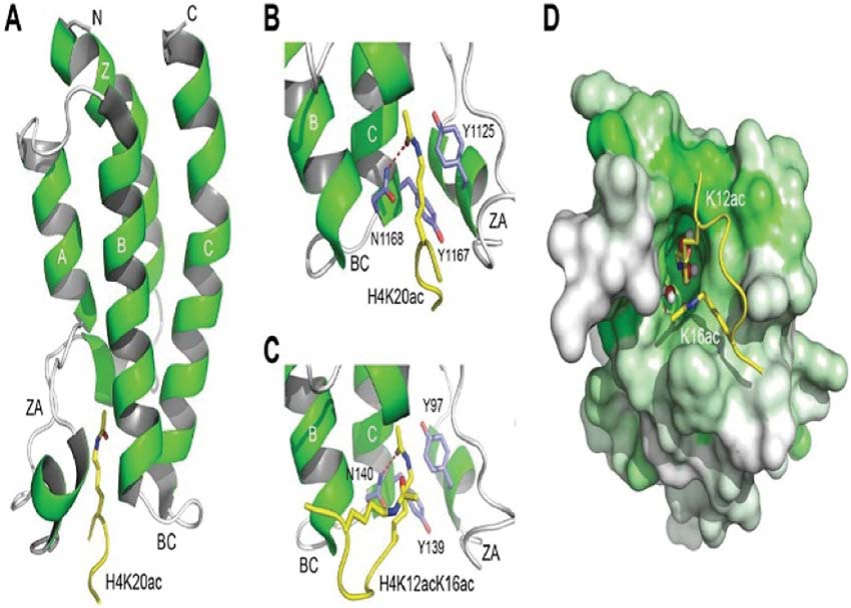
The structural features of the bromodomain as the acetyl-lysine binding domain.

The bromodomains consist of about 110–120 residues and are structurally conserved. The BDs are present in many chromatin-associated factors, including nuclear histone acetyltransferases (HATs), chromatin remodelling factors, and bromodomains and extra terminal (BET) domains family nuclear proteins (Fig. 4).^57^

The dynamic role of lysine acetylation is, to some extent, attributed to the bromodomain (BRD), which is the only protein domain whose conserved activity is to function as an acetyl-lysine binding domain.^42,57^

Several BrD-embracing proteins have been depicted incriminating during disease processes such as cancers, inflammation, and viral replication.^42,59–62^ In recent years, inhibitors of BrDs based on small molecules have allowed many chemical biology-based investigations for processes of BrDs and resolutely recommend that they can be legitimate drug targets in numerous diseases of human.^42,62,63^

In the early 1990s, the transformatively preserved pattern of the bromodomain family was distinguished initially in the *Brahma* gene of *Drosophila melanogaster*.^64^

**Fig. 5:**
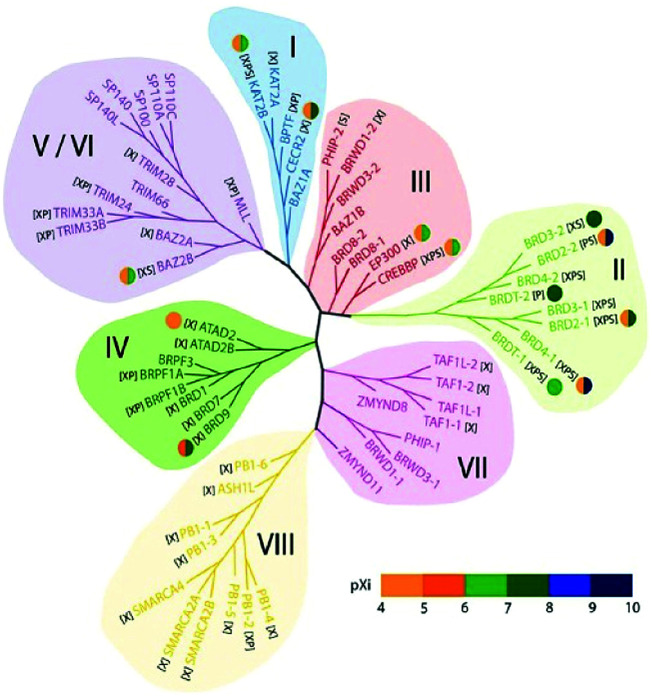
Structure-based phylogeny of the human bromodomains and their inhibitors.

In the human proteome 46 different proteins having a total of 61 bromodomains as per the studies reported.^65^ Based on their structural arrangement they are grouped into eight subfamilies (Fig. 5).^53,65^

These 46 varying proteins have total 61 BRDs, existing as co-regulators in transcription and in enzymes that do chromatin modification, such as HATs and HAT connected proteins (GCN5, PCAF, BRD9),^66,67^ chromatin remodelling complexes that are ATP-dependent (BAZ1B),^67^ helicases (SMARCA),^69^ SET domain-containing methyl-transferases (MLL and ASH1L),^70,71^ co-activators of transcription (TRIM/TIF1)^72^ and mediators (TAF1),^73^ nuclear scaffolding proteins (PB1)^74^ and the BET family (Table 1).^69,75^

**Table 1:** Human BrD-containing Proteins [80] (Including of Isoforms of BRPF1, SMARCA2, SP110, and TRIM33)

Name	Synonyms	Name	Function	# BrDs
ASH1L	ASH1, KMT2H, KIAA1420	Absent small and homeotic disks protein 1 homolog	Histone-lysine methyltransferase	1
ATAD2	ANCCA	ATPase family AAA domain-containing protein 2	Transcriptional regulator	1
ATAD2B	KIAA1240	ATPase family AAA domain-containing protein 2B	Unknown	1
BAZ1A	ACF1, WCRF180, hWALp1	Bromodomain adjacent to zinc finger domain protein 1A	Chromatin-remodelling factor	1
BAZ1B	WBSC10, WBSCR10, WBSCR9, WSTF	Bromodomain adjacent to zinc finger domain protein 1B	Tyrosine-protein kinase; transcriptional regulator	1
BAZ2A	KIAA0314, TIP5	Bromodomain adjacent to zinc finger domain protein 2A	Transcriptional repressor	1
BAZ2B	hWALp4, KIAA1476	Bromodomain adjacent to zinc finger domain protein 2B	Unknown	1
BPTF	FAC1, FALZ	Bromodomain and PHD finger- containing transcription factor	Chromatin-remodelling factor	1
BRD1	BRL, BRPF2	Bromodomain-containing protein 1	Transcriptional regulator	1
BRD2	KIAA9001, RING3	Bromodomain-containing protein 2	Transcriptional regulator	2
BRD3	KIAA0043 RING3L	Bromodomain-containing protein 3	Transcriptional regulator	2
BRD4	HUNK1	Bromodomain-containing protein 4	Transcriptional regulator	2
BRD7	BP75, CELTIX1	Bromodomain-containing protein 7	Transcriptional regulator	1
BRD8	SMAP, SMAP2	Bromodomain-containing protein 8	Transcriptional regulator	2
BRD9		Bromodomain-containing protein 9	Unknown	1
BRDT		Bromodomain testis-specific protein	Chromatin-remodelling factor	2
BRPF1	BR140, Peregrin	Bromodomain and PHD finger- containing protein 1	Transcriptional activator	1
BRPF3	KIAA1286	Bromodomain and PHD finger- containing protein 3	Transcriptional regulator	1
BRWD1	C21 or f107, WDR9	Bromodomain and WD repeat-containing protein 1	Chromatin remodelling factor	2
BRWD3		Bromodomain and WD repeat-containing protein 3	JAK/STAT signalling	2
CECR2	KIAA1740	Cat eye syndrome critical region protein 2	Chromatin remodelling factor	1
CREBBP	CBP, KAT3A	CREB-binding protein	Histone acetyltransferase	1
EP300	P300, KAT3B	E1A-associated protein p300	Histone acetyltransferase	1
KAT2A	GCN5, GCN5L2, HGCN5	General control of amino acid synthesis protein 5-like 2	Histone acetyltransferase	1
KAT2B	PCAF	P300/CBP-associated factor	Histone acetyltransferase	1
MLL	KMT2A, ALL1, CXXC7, HRX, HTRX, MLL1, TRX1	Myeloid/lymphoid or mixed-lineage leukaemia	Histone methyltransferase	1
PB1	PBRM1, BAF180	Polybromo-1	Transcriptional regulator	6
PHIP	WDR11	PH-interacting protein	Insulin signalling	2
SMARCA2	BAF190B, BRM, SNF2A, SNF2L2	SWI/SNF-related matrix-associated actin-dependent regulator of chromatin subfamily A member 2	Chromatin remodelling factor	1
SMARCA4	BAF190A, BRG1, SNF2B, SNF2L4	SWI/SNF-related matrix-associated actin-dependent regulator of chromatin subfamily A member 4	Chromatin remodelling factor	1
SP100		Nuclear autoantigen Sp-100	Transcriptional regulator	1
SP110		Sp110 nuclear body protein	Transcriptional regulator	1
SP140	LYSP100	Nuclear body protein SP140	Transcriptional regulator	1
SP140L	LOC93349	Nuclear body protein SP140-like protein	Unknown	1
TAF1	BA2R, CCG1, CCGS, TAF2A, TAF(ii)250	Transcription initiation factor TFIID subunit 1	Transcription initiation	2
TAF1L	TAF(ii)210	Transcription initiation factor TFIID subunit 1-like	Transcription initiation	2
TRIM24	RNF82, TIF1, TIF1α	Transcription intermediary factor 1-alpha	Ubiquitin E3 ligase, transcriptional regulator	1
TRIM28	KAP1, RNF96, TIF1β	Transcription intermediary factor 1-beta	SUMO E3 ligase, transcriptional regulator	1
TRIM33	KIAA1113, RFG7, TIF1γ	Transcription intermediary factor 1-gamma	Ubiquitin E3 ligase, transcriptional regulator	1
TRIM66	C11orf29, KIAA0298	Tripartite motif-containing protein 66	Transcriptional repressor	1
ZMYND8	KIAA1125, PRKCBP1, RACK7	Zinc finger MYND domain-containing protein 8, protein kinase C-binding protein 1	Transcriptional regulator	1
ZMYND11	BS69	Zinc finger MYND domain-containing protein 11	Transcriptional repressor	1

In the first subfamily (I) proteins having acetyl-transferase P300/CBP-associated factor (PCAF),^67^ amino-acid synthesis general controller 5-like 2 (GCN5L),^67^ Fatal Alzheimer antigen (FALZ)^68^ a transcription factor, and cat-eye syndrome chromosome region 2 (CECR2)^76^ a chromatin remodelling factor all included and present in the nucleus (Table 1).

The subfamily (II) carries bromo and extra terminal (BET) proteins of BRDs, that have a common structural arrangement holds two N-terminal BRDs exhibiting high levels of sequence sustention and also have an extra-terminal (ET) domain and anomalous C-terminal recruitment domain.

BRD2,^76^ BRD3,^77^ BRD4,^78^ and BRDT^79^ are the four proteins that are included in this subfamily. Intriguingly BET proteins during mitosis recruited on the transcription starting sites^80–82^ and BRD4, a BET protein has been reported to lead the positive transcription elongation factor (P-TEFb) utilizing specific towards the C-terminus towards the site of transcription.^54^

The 8B (BRD8B)^83^ containing transcription regulatory bromodomain, binding protein (CREBBP) and E1A binding protein p300 (EP300)^84^ having HAT enzymes, the c-terminal domain of chromatin remodelling factors WD repeat domain 9 (WDR9 domain 2),^85^ adjoining to zinc finger domain 1B (BAZ1B)^68^ bromodomain, bromodomain-containing protein messing up in leukaemia (BRWD3 domain 2)^86^ associated with the C-terminal domain of the JAK/STAT pathway and pleckstrin homology domain interacting protein (PHIP domain 2)^54,87^ connected with the C-terminal domain of the insulin signalling are members of subfamily III of BRDs.

In the subfamily IV bromodomain-containing protein 7 (BRD7)^88^ a transcription regulator, bromodomain-containing protein 1 (BRD1)^78^ and (BRPF1)^89^ composing of PHD finger-containing protein 1, (ATAD2),^90^ having two AAA domain-containing protein, along with the KIAA1240 protein (KIAA1240) (BRD9) having the KIAA1240 protein (KIAA1240) and (BRPF3)^54^ that has the bromodomain and PHD finger containing protein 3.

In subfamily V the transcription repressor tripartite motif-66 (TRIM66),^91^ the tripartite motif-33(TRIM33),^92^ the regulator of transcription, transcription intermediary factor 1 (TIF1),^93^ transcription regulators nuclear auto-antigen Sp-100 (SP100),^94^ antigen Sp-110 (SP110)^95^ and SP140 nuclear body protein (SP140),^96^ along with the SP140-like protein (LOC93349), transcription repressing bromodomain contiguous to zinc finger domain 2A (BAZ2A)^97^ and 2B (BAZ2B)^54,98^ are categorized in human BRDs.

In subfamily VI of human BRDs acetyl-lysine related interactions have not been found in connection to histone or any other proteins. Subfamily VI has histone methyl-transferase myeloid/lymphoid or mixed-lineage leukaemia (MLL)^99^ and transcription co-regulating tripartite motif-28(TRIM28).^54,100^

The transcription repressing zinc finger MYND domain having 11 protein (ZMYND11),^101^ transcription initiating factors RNA polymerase II TATA box binding protein (TBP)-associated factor (TAF1)^102^ TAF1-like (TAF1L),^103^ and the N-terminal BRDs for chromatin remodelling factor WD R9 (WDR9 domain 1),^85^ the JAK/STAT pathway connected with bromodomain based protein, disorganized during leukaemia (BRWD3 domain 1)^86^ and insulin signal relevant pleckstrin homology domain interacting protein (PHIP domain 1)^54,87^ have been grouped in subfamily VII.

Human bromodomain subfamily VIII the last group carries the methyl-transferase ash1 (absent, small, or homeotic)-like (ASH1L),^71^ chromatin remodelling factor SWI/SNF linked chromatin regulator a2 (SMARCA2)^104^ that is actin-based and associated with the matrix, chromatin regulator a4(SMARCA4)^105^ along with the Polybromo 1 (PB1).^73,106^

### BET bromodomains

The family of bromodomain and extra terminal (BET) proteins has been deeply studied. The family includes BRD2, BRD3, BRD4, and BRDT. All of these are ubiquitously expressed, however, BRDT is only expressed in testis.^53^

In cancer, deep associations of BET proteins have been there as they promptly regulate several cancer-related gene expressions, such as c-MYC.^53,107^

**Fig. 6:**
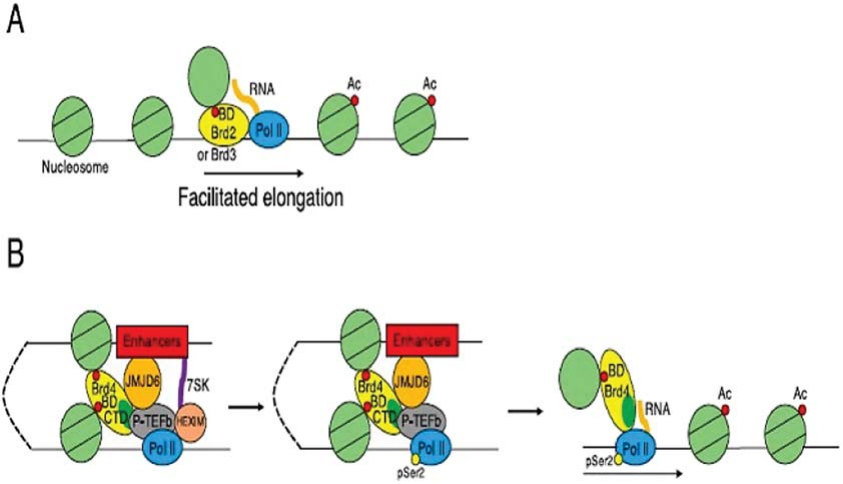
Transcriptional control by BET proteins.

These BET proteins also serve in the regulation of the cell cycle. BRD4 is crucial for the regulation of gene expressions in M to initial G1 phase progression, whereas BRD2 prepares a scaffold on the chromatin that raises the indispensable cell-cycle transcription regulation genes E2F1 and E2F2 (Fig. 6) (Table 2).^53^

**Table 2:** Functions of mammalian bromodomain and extra-terminal domain (BET) proteins

BET protein	Functions	References
BRD2	− Promotion of E2F-dependent cell cycle progression in HeLa and HEK293 cells	110 and 111
	− Closure of the neural tube in mouse embryos	112 and 113
	− Maintenance of the number of GABAergic neurons in the neocortex and the striatum of mice	114
	− Assist of transcription in hyperacetylated chromatin (Property of histone-chaperone)	77
	− Transcriptional activation of HOXA11 and D11 in HEK293 cells	115
	− Enhancement of GATA1-mediated erythroid gene activation	116
	− Interaction with LANA of KSHV that mediates episomal replication and persistence of viral genomes	117 and 118
BRD3	− Assist of transcription in hyperacetylated chromatin (Property of histone-chaperone)	77
	− Transcriptional activation of HOXB3, B4, B5, B6, C8, C9, C10, A3, A5, A6, and A7 in HEK293 cells	115
	− Enhancement of GATA1-mediated erythroid gene activation	116
	− Carcinogenesis induced by BRD3-NUT fusion protein	119
BRD4	− Stimulation of G2/M transition in HeLa cells	80
	− Cell cycle progression in P19 embryonal carcinoma cells	81
	− Maintenance of inner cell mass in mouse blastocysts	120
	− Transcriptional activation of Nanog required for maintaining the pluripotency of ES cells	121
	− Release from a pause in transcription elongation	122 and 123
	− Assist of transcription in hyperacetylated chromatin (Property of histone-chaperone)	124
	− Transcriptional activation of c-Myc and Klf4 in NIH3T3 cells	124
	− Transcriptional activation of HOXB2, B3, B4, B5, B6, B7, B8, A4, and C5 in HEK293 cells	115
	− Transcriptional regulation of genes involved in learning and memory in mice	125
	− Enhancement of INF-induced gene transcription	126
	− Signal transducer of the cellular response to oxidative stress	127
	− Prevention of splicing inhibition in heat stress-induced cells	128
	− A gene bookmark for transcriptional reactivation in post-mitotic cells	129 and 130
	− Carcinogenesis induced by BRD4-NUT fusion protein	119 and 131
	− Interaction with LANA of KSHV that mediates episomal replication and persistence of viral genomes	132 and 133
	− Tethering of BPV genome to host mitotic chromosomes	134
	− Transcriptional regulation of E2 that mediates episomal maintenance and DNA replication of HPV genome	135–137
BRDT	− Transcriptional regulation of genes responsible for meiotic progression during spermatogenesis	126
	− Splicing machinery in testicular cells	138
	− Chromatin remodelling in MEL, 3T3, and COS7 cells	139–141
	− Histone replacement at post-meiotic stages during spermatogenesis	125

**Fig. 7:**
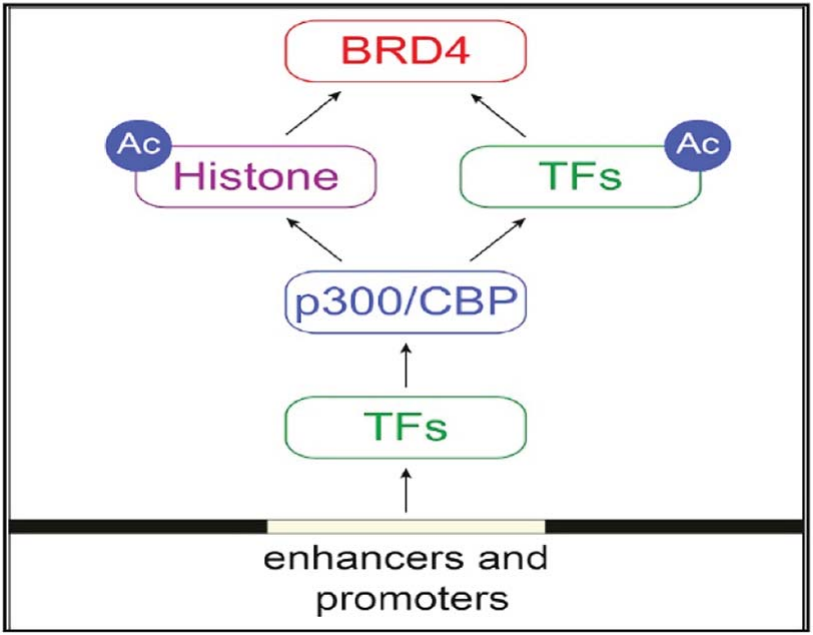
The BET protein BRD4 required for the functional output of an ensemble of lineage-specific transcription factors.

**Fig. 8:**
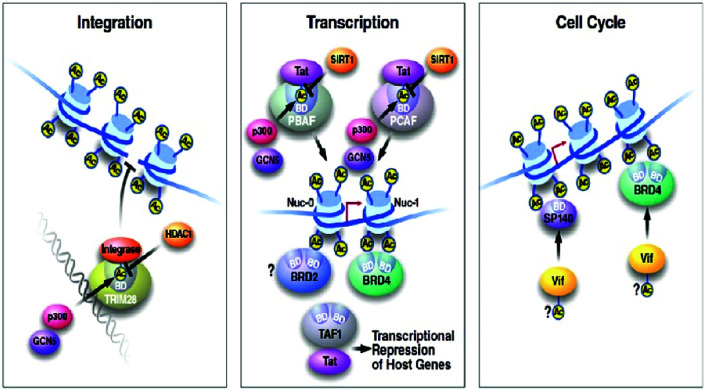
Binding to acetylated histones.

**Fig. 9:**
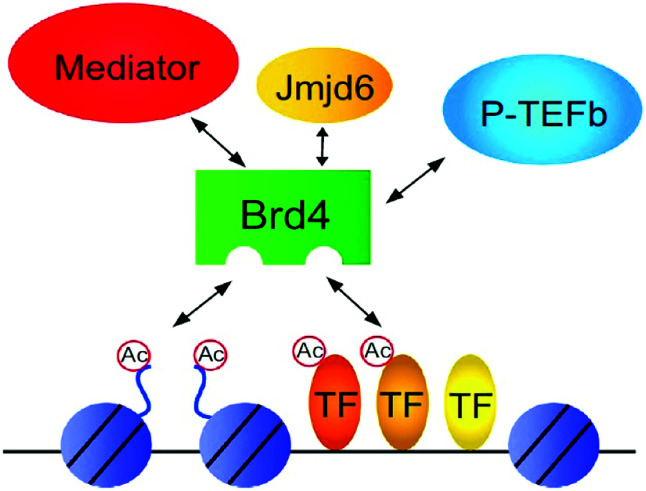
Protein–protein Interactions.

**Fig. 10:**
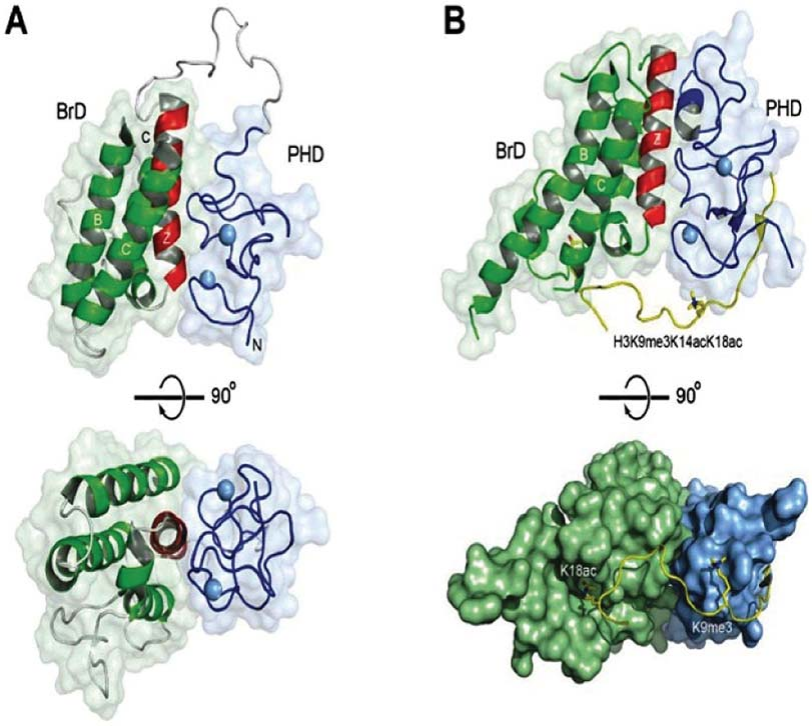
Structures of tandem modules of epigenome reader domains.

BRD4 is a universal gene transcription regulator so the inhibition of BRD4 would be predicted to attempt universal down-regulation of gene functionality. Inhibition of BRD4 is of prime importance as it is regulating several hundred genes essential for tumorigenesis (Fig. 7–10).^53^

### Other bromodomain proteins (BRD9 and TRIM24)

As transcriptional co-activators such as tripartite motif-containing proteins (TRIMS) and TBP-associated factors (TAFs) bromodomains show their presence.^53^

An identified chromatin-remodelling BAF SNF/SWI complex component is (BRD9) bromodomain-containing protein 9. Less information has been available about the functionality of it however essentiality of it in cancer has been documented. Recently, in a study, it has been described that for sustaining MYC transcription AML cells need BRD9 and through that proliferation has been increased. Almost similar to BTD9 bromodomain-containing protein 7 (BRD7) is also a subunit of PBAF SWI/SNF. In several reports, it has been documented that as a tumour suppressor gene, BRD7 either partially or completely downregulate several cancers such as small-cell lung cancer, ovarian, colorectal and breast cancers, endometrial carcinoma, and hepatocellular carcinoma where it is part of BRCA1.^54^

An epigenetic way has been proposed to attempt CRPC for bromodomain and extra-terminal (BET) family protein suppression. In tumour models of CRPC, growth retardation has resulted through BET inhibitors.^142–144^

The selectively binding to acetylated lysine is done by bromodomain family proteins, the third type of proteins that do epigenetic regulation and based on that acts as “readers” of the acetylated lysine.^58^

A subset of 46 bromodomain-containing proteins available only in the genome of human.^145^ Bromodomain containing protein 2 (Brd2), Brd3, Brd4, and testis-specific protein (BrdT) all four together constitute BET protein family.

BRD4 through binding with Kac residues present on histone tails regulates gene expression. This regulation is done by recruitment of positive transcription elongation factor *b* (*p*-TEFb) on (RNA pol II) RNA polymerase II enzyme having phosphorylation.^108,109^

BET proteins, especially Brd4 deregulated and this has been involved in diverse diseases, such as cancer formation and progression. Zuber *et al.* disclosed that in the preservation of c-Myc gene expression and stimulation of deviant self-renewal of AML cells, Brd4 has a vital role.^146^

### HDAC family

The enzyme class that has histone deacetylases (HDAC), doing acetyl groups (O=C–CH_3_) removal from an ε-*N*-acetyl-lysine amino acid located on histone, permits tighter DNA wrapping by histones.^52^

From yeast originated enzymes based on their homology of sequence and organization of domain, HDAC thus grouped into four classes, class I, II, III, and IV.^244^

The class of HDACs that consists of a zinc-dependent active site and can be controlled by trichostatin A (TSA) is designated as a “classical” family of HDAC that has class I, II, and IV. On the other hand, class III of the HDAC enzyme family have sirtuins that can be impacted by TSA as they are NAD^+^-dependent proteins.^147^ From the yeast-based reports, the homologous of these three classes of HDACs have been names as reduced potassium dependency 3 (Rpd 3) that correlated with the group I, class II connected with histone deacetylase 1 (HDAC1), and class III enzymes interrelated with silent information regulator (Sir2). With only one isoform (HDAC11) that is not truly homologous with any of Rpd3 or HDAC1 enzymes of yeast so HDAC11 has been assigned to its class IV. The class III enzymes have the deviating mode of action and are NAD^+^-dependent since other classes of enzyme HDACs are dependent on Zn^2+^, a cofactor.^148,149^

### Present epigenetic target drugs (inhibitors and degraders)

The resultant effect of Brd4 knockdown through shRNAs or by small-molecule based pharmacologic suppression of Brd4 showed consecration of terminal differentiation and eradication of leukemic stem cells. It has also been reported about effective anti-leukaemia potential in numerous AML cell lines and initial patient-derived cells from human.^146,150^

The expansion of tamoxifen-resistive breast cancer cells can effectively be retarded by BET protein suppression as reported by Malley *et al.*^151^ In melanoma types of cancer growth, Brd4 has been found highly-strung even at initial and metastatic tissues that have melanoma phase. Prompt inhibition of key cell-cycle genes, having SKP2, ERK1, and c-Myc, can be achieved through Brd3 inhibitor therapy. *In vitro* melanoma cell spread and *in vivo* tumour development and metastatic representation has been effectively attenuated through Brd4 inhibitor therapy. The impactive anti-leukemic properties of brd4 inhibitor mediated suppression have been epitomized by the silencing of Brd4 on an individual basis.^152^

Brd4 silencing can further be recognized Brd4 as a target for therapeutic designing and leads to emphasis discoveries for validation of Brd4 as a druggable target. Two immensely homologous bromodomains on amino-terminal loci absolute assignment of nucleosomes by attaching at distinct acetylated lysines (Kac) on histone tails are critical for the functioning of BET proteins.^68^

### Brd4 inhibitors

Based on interactive modules between BDs and inhibitors two classes of Brd4 inhibitors constituted: monovalent and bivalent. Each bromodomain of Brd4 protein has been separately targeted for binding by valent type Brd4 inhibitors while both bromodomains concurrently joined with bivalent Brd4 inhibitors as they have such proficiency.^152^

**Fig. 11:**
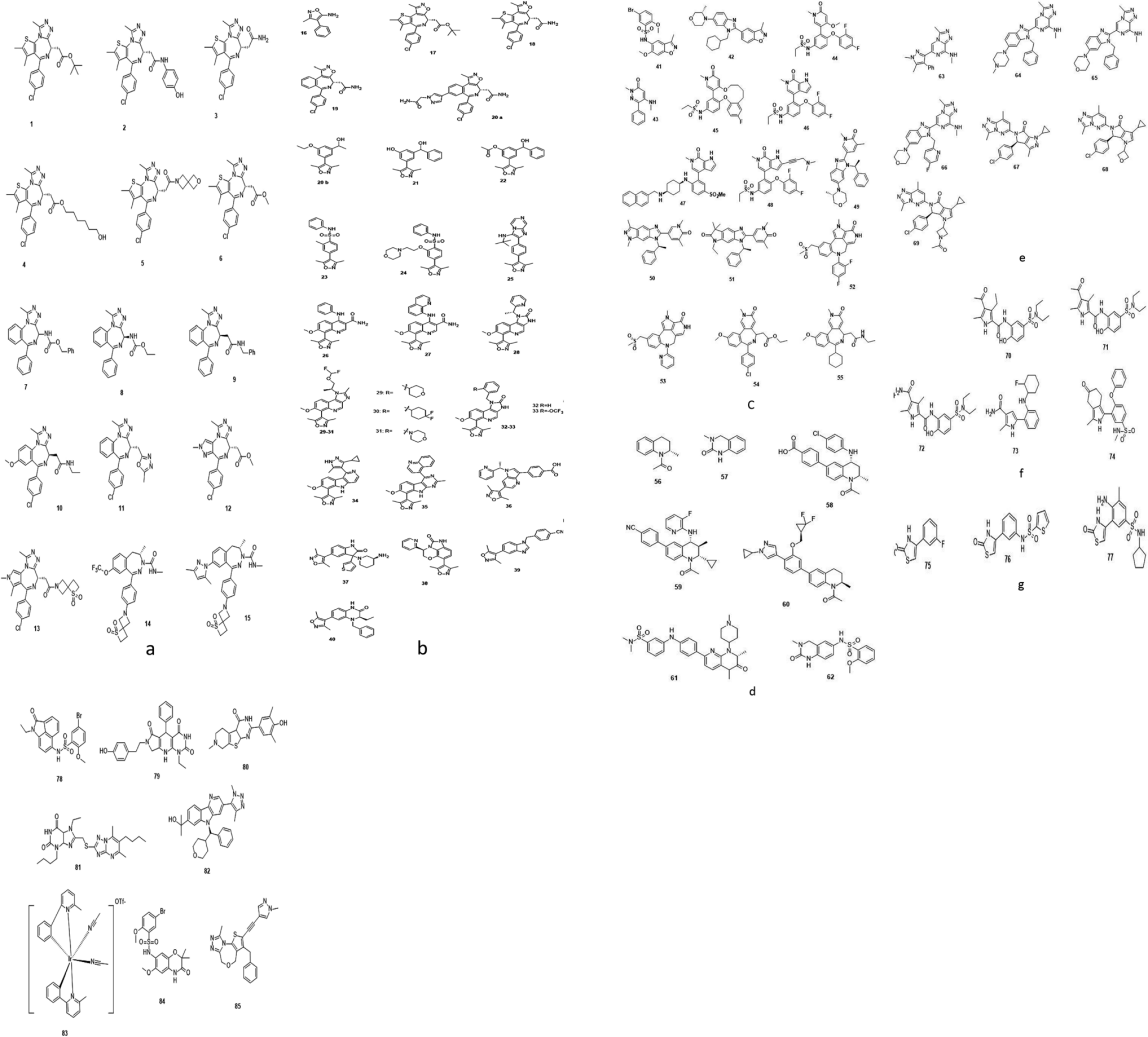
Monovalent Brd4 inhibitors.

(a) Monovalent Brd4 inhibitors Fig. 11(a–h).

(i) Triazolo azepine-based Brd4 inhibitors.^153–165^

(ii) Isoxazole-based Brd4 inhibitors.^70,106,142,153–155,166–179^

(iii) Pyridone-based Brd4 inhibitors.^174,180–185^

(iv) Tetrahydroquinoline-based Brd4 inhibitors.^143,186–191^

(v) Triazolo pyrazine-based Brd4 inhibitors.^192–194^

(vi) 4-Acyl pyrrole-based Brd4 inhibitors.^195–197^

(vii) 2-Thiazolidinone-based Brd4 inhibitors.^198^

(viii) Other reported inhibitors.^199–204^

**Fig. 12:**
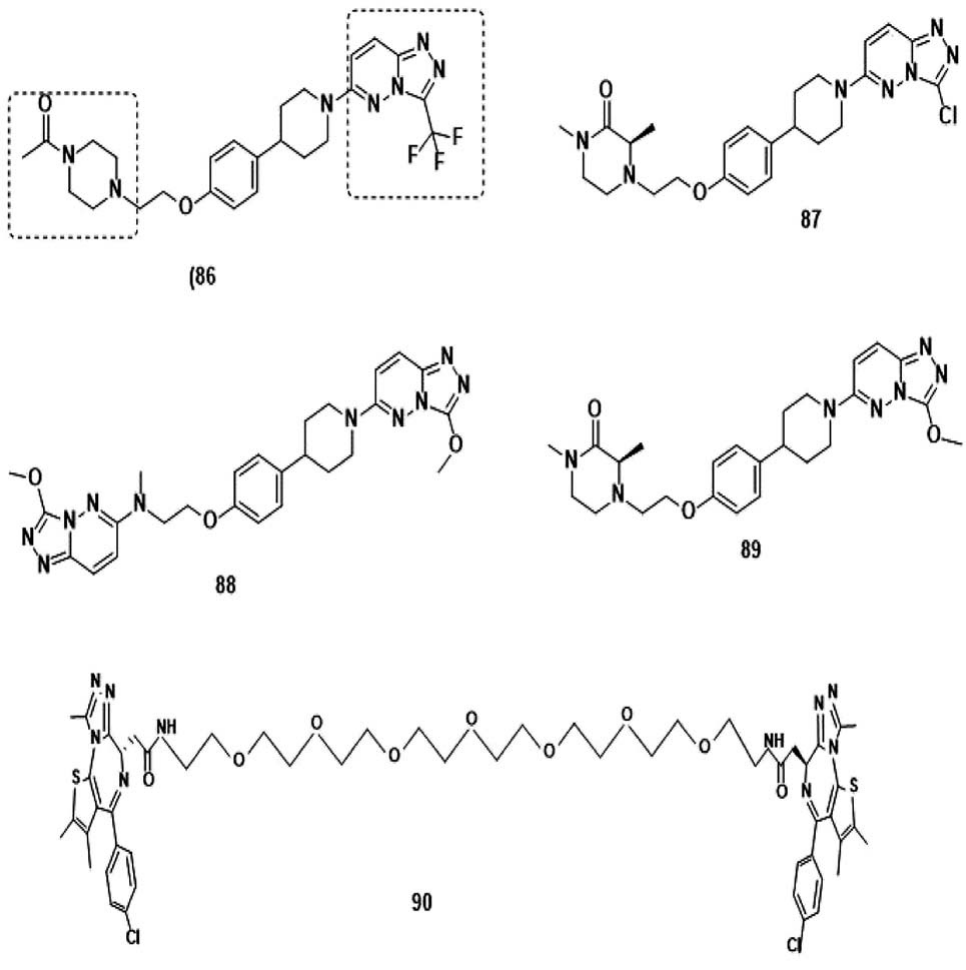
Bivalent Brd4 inhibitors.

(b) Bivalent Brd4 inhibitors (Fig. 12).^205–210^

**Fig. 13:**
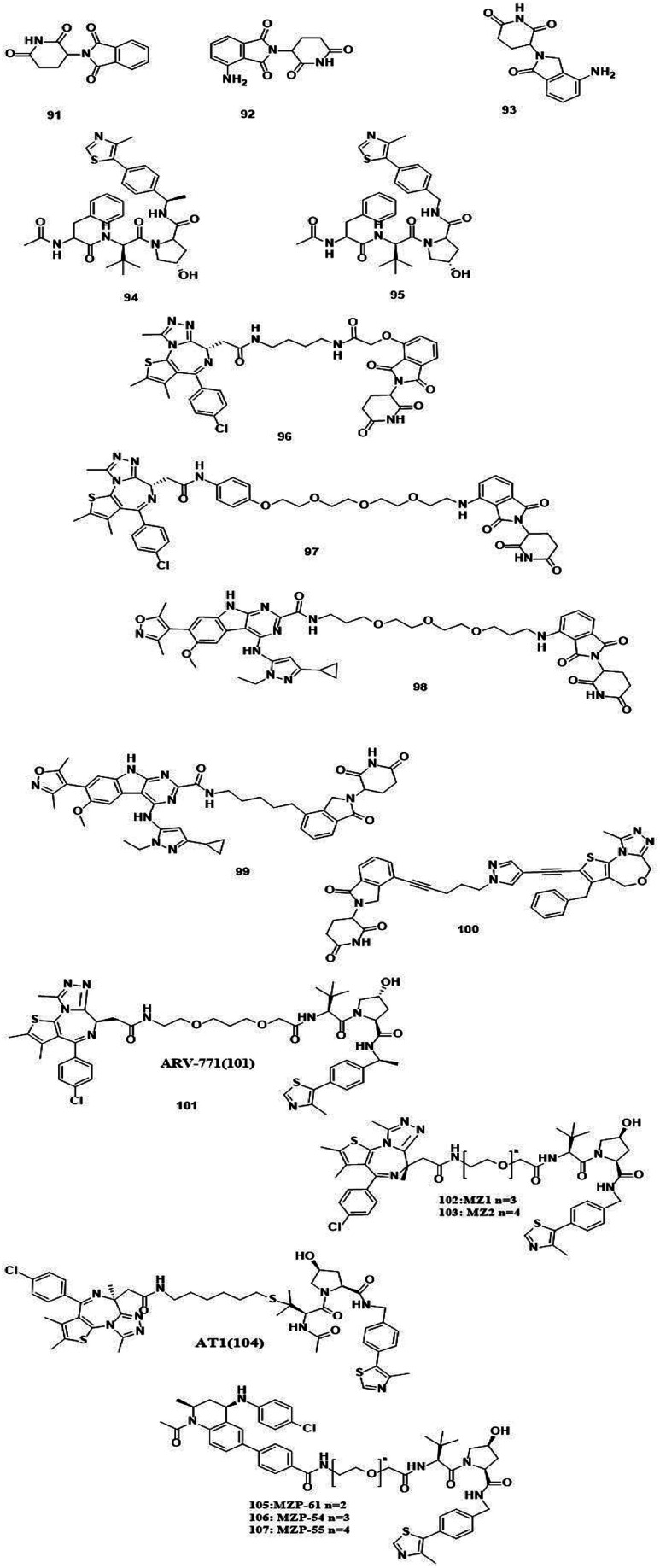
Brd4 degraders.

### Brd4 degraders (Fig. 13)

Based on recent pieces of work it is reported that Brd4 inhibitors prompt settlement in terms of Brd4 protein accumulation during several types of cancers such as lung and prostate cancer and Burkitt's lymphoma due to unimpacted c-Myc retardation, tuned apoptotic initiation, and antiproliferative processes,^211,212^ although Brd4 inhibitors have shown their auspicious capabilities in numerous C-Myc-driven malignancies. Besides, drug resistivity against triazolo azepine based Brd4 inhibitors I-BET762 and (+)-JQ1 has also been demonstrated.^213^

In consideration of the channel between cancer and Brd4 expression, Brd4 has been recognized as a bright therapeutic target in various kinds of malignancies.^214,215^ Notable attempts have been invented to flourish pharmacological inhibitors of Brd4 and several Brd4 inhibitors have upgraded to clinical and preclinical assessment.^216,217^

### HDAC inhibitors

Valproic acid-like histone deacetylase inhibitors (HDIs) have been utilized since long as mood-stabilizing agents and antiepileptics drugs in neurological disorders and psychiatric therapeutics.^218,219^

Histone deacetylase inhibitors (HDIs) have a long history of use in psychiatry and neurology as mood stabilizers and anti-epileptics, for example, valproic acid.^218,219^ At the current time phase, numerous endeavours have been done to establish HDIs as a cancer remedy. In 2006, Vorinostat (SAHA) got permission to use in the treatment of cutaneous instances in patients that have concomitant T cell lymphoma and have been failed to be cured in earlier therapies.^220,221^

Another HDI, istodax has been endorsed in 2009 for the patients with CTCL4. The exact mode of actions of these molecules that may trade is uncertain, perhaps epigenetic pathways are intended. Further, the effectiveness of valproic acid on the latent pools of HIV that are influenced in persons is under clinical investigation.^222^ HDIs at present are also being reviewed as chemosensitizers for radiation or cytotoxic chemotherapy, or in companionship with DNA methylation inhibitors-based harmony *in vitro* phase. Non-histone proteins linked to acetylation and can shift the degree of acetylation can be affected by HDI molecules and thus step up or down their activity.

SIRT2, as a member of the Class III HDAC family, is an NAD^+^-dependent enzyme. It could interact with a range of proteins and then remove acyl groups which played an important role in many cellular functions.^223^

### The development of PROTACs

During the last two decades, a good sort of work has been done in the field of PROTAC development. Initially described by Craig Crews and Ray Deshaies in 2001, MetAP-2 was degraded by protein targeting chimeric molecule 1 (Protac-1) which recruited MetAP-2 to SCF binding phospho-peptide and small-molecule ovalicin.^224^ It is in 2004 that the first cell-permeable PROTAC was found which induced androgen receptor degradation.^225^ Subsequently, PROTACs have entered into a phase with rapid development, and some other kinds of proteins including MetAP-2 and estrogen receptors were displayed to be knocked out in a range of cell lines.^226^ However, peptide-based PROTACs show shortcomings on unstable peptide bonds, high molecular weight, and poor cell penetration. The disadvantages mentioned above make it poor pharmaceutical candidates. To overcome these weaknesses of PROTACs including peptide 8,^227^ small-molecule PROTACs were designed and synthesized and are more easily absorbed than peptides by the human body. MDM2, cIAP, VHL, and cereblon were selected as E3 ligases.^228^

A peptide moiety in the form of E3 ligase ligand was present in all first-generation PROTACs. However, due to limited physicochemical properties including less cell permeability, little intracellular stability, and poor applicability in therapeutic development as a chemical molecule are the resultant impacts of a high peptide containing all PROTACs of first-generation.^261^

It was in 2008 when Crews described all-small molecule PROTAC having a heterobifunctional ability that made up of a PEG-based linker, an androgen receptor (AR) ligand, and an MDM2 ligand (nutlin) all together can initiate ubiquitination and then proteasome based degradation.^229^ Nutlin (MDM2 ligand) is a group of imidazoline derivatives, which bind to MDM2 for blocking the interaction between MDM2 and p53.^230^ However, promising as a critical first step away from peptide-based PROTACs, this initial small molecular degradation inducer is less effective than its peptide analogues. Hashimoto research group reported bestatin-based PROTACs binding cIAP1 could induce the degradation of nuclear receptors including AR, ER, and retinoic acid receptor.^231^ It is a pity that this kind of PROTACs presented serious off-target effects. The study from Science found that thalidomide binds to E3 ligase cereblon to induce degradation of ikzf1 and IKZF3, suggesting thalidomide and its derivatives as initial ligands targeting E3 ligase CRBN. ARV-825, consisting of OTX015 linked to pomalidomide *via* alkyl group, degraded almost complete BRD4 protein at 10 nm within 6 h.^232^ Since 2015, the publication of a series of papers on small molecular VHL-based PROTACs reached its peak little by little.^233,234^ PROTAC technology has been applied by several drug discovery labs. Yale University licensed the PROTAC technology to Arvinas in 2013. What excites people is that the U.S. Approval for the first phase clinical trial of Arvinas has been obtained from FDA to investigate whether ARV110 can be used as a therapy for patients suffering from metastatic prostate cancer that has castration-resistivity. The trial, scheduled to begin by April, will investigate the safety and tolerability of ARV110 in mCRPC patients whose disease progressed after being treated with a minimum of two standards of care therapies.^235^

### Application of PROTACS for epigenetic targets

Besides downregulation of targeted protein, selectively initiated target protein degradation emerged as a novel strategy in drug discoveries.^236,237^

By designing proteolysis-targeting chimeras (PROTACs), to degrade target protein is one of the convicting approaches at present and has highly attracted medicinal chemists and pharmaceutical firms.^236,238–240,244^

Proteins marked for proteasomal degradation are tagged *via* covalent attachment of ubiquitin to surface lysine.^241,242^ Inherited or acquired diseases are often based on abnormal protein functioning, which is currently targeted using a predominantly occupancy-based pharmaceutical strategy; inhibitors bind to disease-implicated proteins and the longer protein function is blocked by inhibitors, the larger the clinical benefit achieved. Therefore, high local inhibitory concentrations (IC90–95) need to be maintained at all times to ensure therapeutic efficacy.^243^

**Fig. 14:**
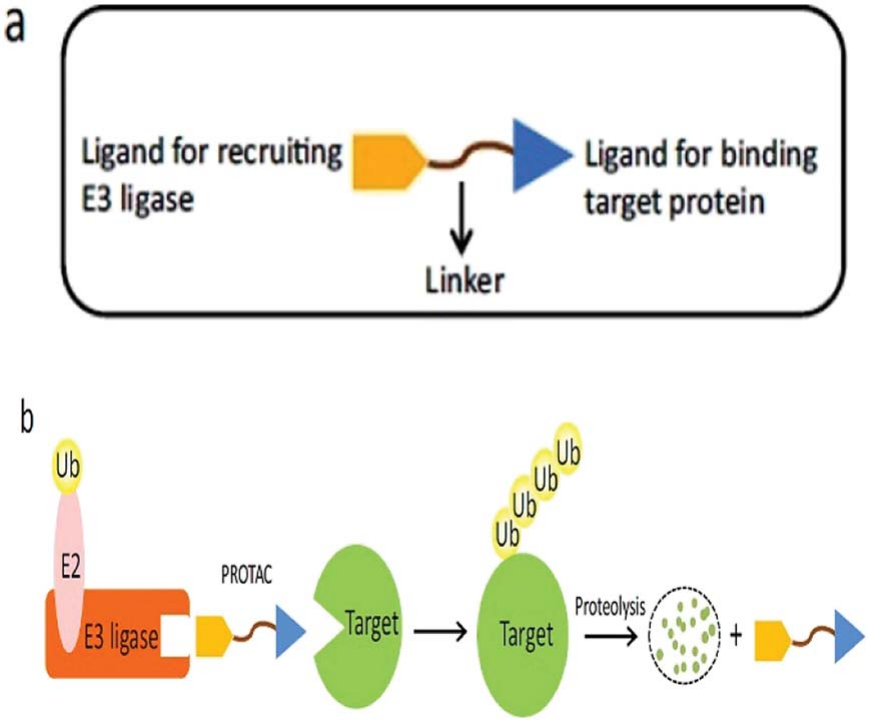
(a) A PROTAC molecule consists of a ligand for recruiting an E3 ubiquitin ligase, a linker, and a ligand binding to the target protein. (b) The PROTAC binds with both the target protein and the E3 ligase simultaneously to induce the formation of a ternary complex. The target protein is then polyubiquitinated and undergoes proteolysis.

A heterobifunctional small compound was initially proposed by Deshaies *et al.*,^244^ about 15 years back. It consists of three subunits, an E3 ubiquitin ligase binder, a target protein-specific ligand, and a linker or connector that is connected with these two mechanisms (Fig. 14a).

Deshaies^244^ and co-workers synthesized the first-ever proteolysis-targeting chimeric molecule (PROTAC), that can hijack the (UPS) for protein degradation on broad-spectrum during post-translational levels.^36^

For the alimentation of cellular homeostasis, one of the essential mechanisms that do protein degradation is UPS. It consists of three enzymes, designated molecules, intracellular target proteins, proteasome, and ubiquitin that is responsible for playing a vital role for numerous biological functions such as signal transduction, genome integrity conservation, tumorigenesis, and cell cycling.^246^

An ATP-incidental enzymatic process, protein ubiquitination, is accomplished by a ubiquitin-activating enzyme (E1), a ubiquitin-conjugating enzyme (E2), and a ubiquitin ligase (E3) (Fig. 1).

Disease-generating proteins are degraded by the UPS hijacking instead of inhibition mechanism as in the traditional way by small-molecule based inhibitors. This contributes to a more advanced potent approach.^249^ Furthermore, as any concerned protein can be targeted by PROTAC, it expressed a more convincing technological aspect in drug discovery as it is not restrained to UPS dependent substrates only.^10^

A tertiary complex is formed during the binding of target protein, PROTAC, and the E3 ligase. In the further event, ubiquitin can be shifted to protein target as recruitment of an E3 ubiquitin ligase has been done and the target protein has been degraded out through the proteasome (Fig. 14b).^243,245,246,248–252^

For treating cancer, protein inhibition has given low preference than oncoprotein degradation through PROTACs in theoretical aspects. At the initial level entire protein removal is likely to be more adequate than its inhibition at its active site as remaining protein structure and domains are yet active or functional, next, PROTACs can act enzymatically for the degradation of any targeted protein, and finally, transcription factors like “undruggable” proteins can also be intended.^253,254^

Sakamoto *et al.*,^36^ has prepared the first-ever PROTAC, that is having phosphopeptide (DRHDpSGLDpSM) imitated form NF-κB inhibitor-α (IκBα) to obtain SCFβ-TrCP E3 ligase; ovalicin (OVA), that can bind covalently to methionine aminopeptidase-2 (MetAP-2) active site (His-231) and ubiquitinated MetAP-2 and a linker that can join phosphopeptide and OVA.^10^

Seven-amino-acid sequence (ALAPYIP), as primitive PROTAC of *in vivo*, substituted the IκBα-phosphopeptide component coupled with artificial ligand (AP21998) for targeting (F36V) FKBP12 proteins.^255^

This minimal amino acid sequencing the hypoxia-inducible factor 1α (HIF1α) can be identified by the von Hippel-Lindau tumour suppressor protein (VHL), a known ingredient of CRL2VHL E3 ubiquitin ligase.^254^

Another advantage is the carboxy terminus of ALAPYIP has an eight-poly-d-arginine tag, that enhances cell permeability and restricts nonspecific proteolysis.^256,257^

The target proteins have been eradicated in the cellular ambiance by cell-permeable PROTACs. The materialization of cell-permeable PROTACs has been a momentous invention in PROTAC technology and accommodates opportunities for *in vivo* disease-causing protein targeting.^10^ This moves towards auspicious administration in the field of cell biology and more precisely for drug development.

Currently, tyrosine kinases,^111^ estrogen receptor α,^112^ CDK9,^113^ Bcr-Abl,^114,115^ and many other kinds of proteins are degraded by PROTACs. An offbeat epigenetics-based therapy for cancers and have been proposed for provocative antitumor efficiency.^152^

At present, for targeting enzymes, regulating proteins, transcription factors, skeleton proteins, *etc.* can be targeted through PROTAC technology.^247,258^

More advantages have been reported by small molecule-based PROTACs beyond peptide-based PROTAC.^259^ Interestingly, small molecule-based PROTAC has better capabilities for utilization as a drug smaller molecule can easily be absorbed in the human body over a peptide.^258^

At present, above 30 proteins were found as a target in disease initiation and progression with primary endeavour over proteins in the therapy of cancer.^10,260,261^

Several combinations are possible for PROTACs as (1) many types and kinds of ligands can be utilized for specific binding with target proteins and to initiate these proteins on the E3 ligase, and (2) over 600 different ligases encoded in the human genome allowing a broad spectrum of PROTACs based drug invention.^10^

**Fig. 15:**
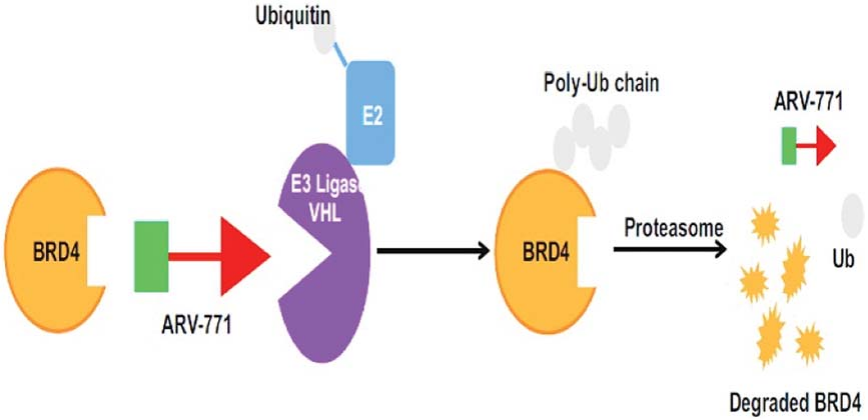
BRD4 PROTAC schematic.

Small-molecule based BET protein degraders have been developed by some researchers no long ago.^262^ “Proteolysis targeting chimeras” (PROTACs), are heterobifunctional molecules *via* trimeric binding complexes permitting ubiquitination and on later stage proteasome-based degradation of target protein (Fig. 15).^244^

A BET protein that utilizes the E3 ligase cereblon (CRBN) ends up in impressive BET degradation and uninterrupted restraint of downstream signalling in cell lines of Burkitt lymphoma, reported by Raina *et al.*^234^

An eminently strong degrader (ARV-825) of the epigenetic regulator BRD4 has been emerged from CRBN-recruiting pomalidomide in mingling with the bromodomain-containing protein 4 (BRD4) inhibitor OTX015.^243,262^

The protein levels of BRD2, BRD3, and BRD4 has been diminished by most impactive BRD PROTAC, dBET1, with less nanomolar efficacy and surpassed JQ1 for activating of apoptosis in cell lines of AML and lymphoma.^243^

In the recent past, degraders for the BRD isoform 7 and 9, which plays a vital role in tumour development have been investigated by Zoppi *et al.*^263^

**Fig. 16:**
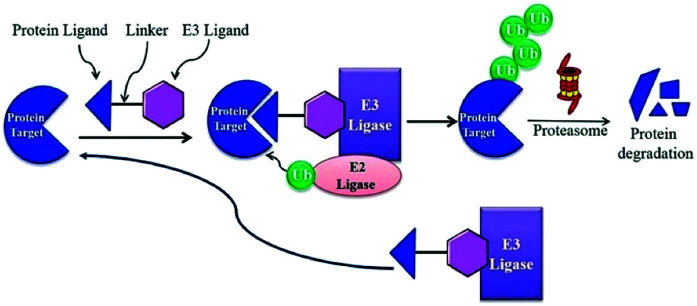
Mechanism of protein degradation by PROTACs.

**Fig. 17:**
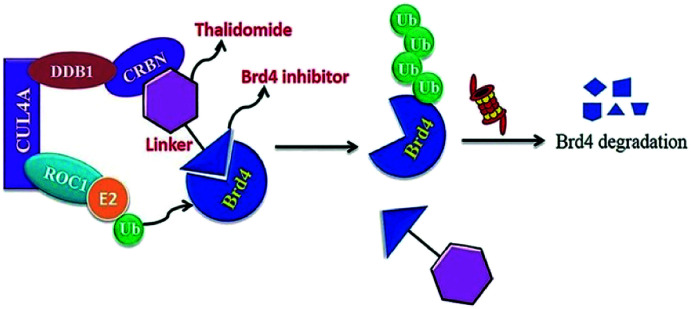
Mechanism of Brd4 degradation by CRL4 CRBN E3-based Brd4 degraders.

**Fig. 18:**
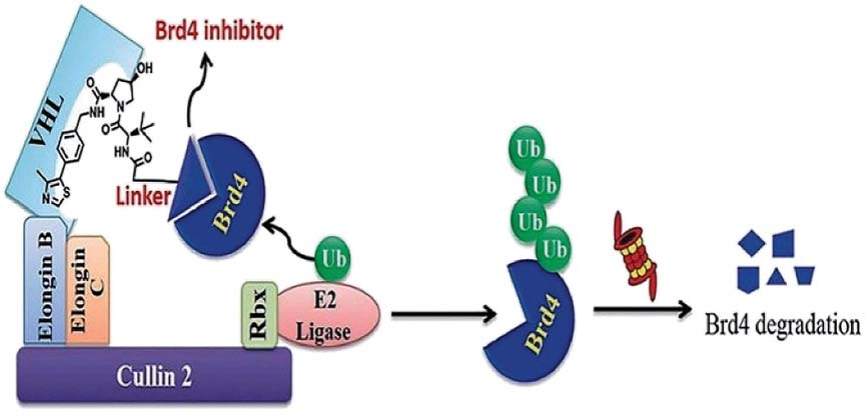
Mechanism of Brd4 degradation by CRL2VHL E3-based Brd4 degraders.

BRD7 inactivation makes tumour cells become targetable for T-cell-mediated killing. Because of this, PROTAC selective for BRD7/9 can be used as a chemical probe to study recognition of target and further possibilities for therapeutic invasion (Fig. 16–18).^264^

**Fig. 19:**
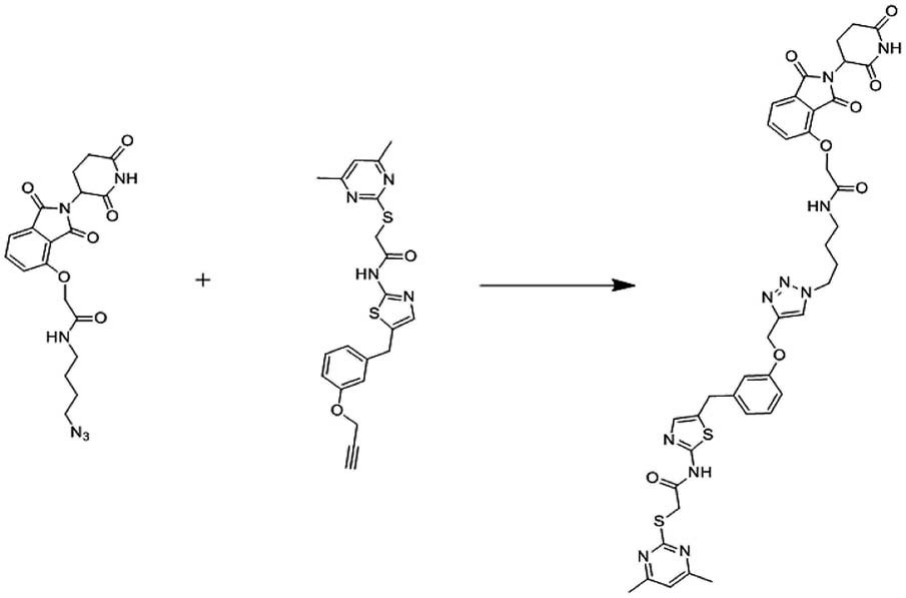
Reported sirt2 PROTAC.

In 2008, Manfred Jung and co-workers first reported chemically induced degradation of Sirtuin 2 by PROTAC based on conjugation SirReals and thalidomide (Fig. 19).^268^

This designed PROTAC (compound Y) presented highly selective SIRT2 inhibitory activity (SIRT2, IC50 = 0.25 μM; SIRT1 and SIRT3, IC50 > μ mM). However, it is unclear whether the degradation of SIRT2 by PROTAC can be used as a strategy for the treatment of tumours.

**Fig. 20:**
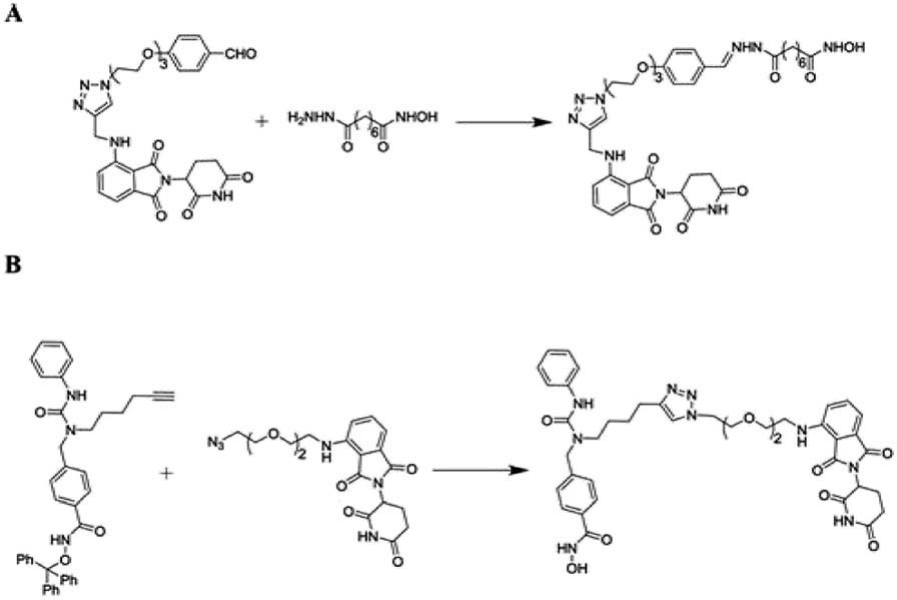
Reported HDAC6 PROTAC.

In humans, an enzyme Histone deacetylase 6 (HDAC6) concealed with the HDAC6 gene is belonging to HDAC family class II and is found in the cytoplasm.^265^ Contradictory to other nuclear histone targeting HDACs, HDAC6 is effective against transcription and translation as it regulates (Hsp90) the heat-shock protein 90 and stress granules (SGs), in accordingly. It is also acknowledged for binding with ubiquitinated proteins with great compatibility and has a role and involution in SG protein formation.^266^ Small molecule-based PROTACs against zinc-dependent HDAC6 associated inhibitors of HDAC in compulsion with pomalidomide have been firstly reported by the Tang research group (Fig. 20A).^267^

And more, better selectivity and potential against HDAC6 are ongoing and will be reported in due course from the author's statement.

Subsequently, Rao designed novel HDAC6-targeting PROTACs connecting HDAC6 inhibitor nexturastat A and pomalidomide by the aliphatic chain (Fig. 20B). Compound X induces HDAC6 degradation at 100 nmol L^−1^ and shows the most potent activities against a range of cell lines. It is gratifying that further functional research of compound X *in vivo* is now underway in his laboratory.

## Conclusions

Cellular functions of BET proteins and their necessity in several malignancies as well as diseases like cardiovascular problems, HIV infection and inflammation have been reported in earlier decades. In addition to functional inhibition by small molecules, Brd4 has been successfully targeted for degradation using PROTACs. Many possibilities have been found in this novel approach of PROTAC development as it proved its selective target degradation abilities, however, some issues such as physicochemical properties, scarce small E3 ligands, are destinations that should be overcome to make PROTACs boost into the clinic.

## Conflicts of interest

There are no conflicts to declare.

## Supplementary Material
